# Dairy Product Consumption and Metabolic Diseases in the Di@bet.es Study

**DOI:** 10.3390/nu11020262

**Published:** 2019-01-24

**Authors:** Ana Lago-Sampedro, Eva García-Escobar, Elehazara Rubio-Martín, Nuria Pascual-Aguirre, Sergio Valdés, Federico Soriguer, Albert Goday, Alfonso Calle-Pascual, Conxa Castell, Edelmiro Menéndez, Elías Delgado, Elena Bordiú, Luis Castaño, Josep Franch-Nadal, Juan Girbés, Felipe Javier Chaves, Sonia Gaztambide, Gemma Rojo-Martínez, Gabriel Olveira

**Affiliations:** 1 Instituto de Investigación Biomédica de Málaga-IBIMA, 29010 Málaga, Spain; anishhh22@gmail.com (A.L.-S.); elehazara@gmail.com (E.R.-M.); sergio.valdes@hotmail.es (S.V.); federicosoriguer@gmail.com (F.S.); gabrielm.olveira.sspa@juntadeandalucia.es (G.O.); 2 UGC Endocrinología y Nutrición, Hospital Regional Universitario de Málaga, 29009 Málaga, Spain; 3 Biomedical Research Network in Diabetes and Associated Metabolic Disorders (Centro de Investigación Biomédica en Red de Diabetes y Enfermedades Metabólicas Asociadas -CIBERDEM-), Instituto de Salud Carlos III, 28029 Madrid, Spain; acallepascual@hotmail.com (A.C.-P.); lcastano@osakidetza.net (L.C.); 19658jfn@comb.es (J.F.-N.); felipe.chaves@uv.es (F.J.C.); mariasonia.gaztambidesaenz@osakidetza.net (S.G.); 4 UGC Endocrinología y Nutrición, Instituto de Investigación Biomédica de Málaga (IBIMA), Universidad de Málaga, 29010 Málaga, Spain; 5 UGCI de Prevención, Promoción y Vigilancia de la Salud, Complejo Hospitalario Universitario Granada, 18016 Granada, Spain; nuria_pascual@hotmail.com; 6 Department of Endocrinology and Nutrition, Hospital del Mar, 08003 Barcelona, Spain; agoday@hospitaldelmar.cat; 7 Department of Endocrinology and Nutrition, Hospital Universitario S. Carlos de Madrid, 28040 Madrid, Spain; 8 Public Health Division, Department of Health, Autonomous Government of Catalonia, 08023 Barcelona, Spain; conxa.castell@gencat.cat; 9 Department of Endocrinology and Nutrition, Hospital Central de Asturias, 33011 Oviedo, Spain; edelangot@gmail.com (E.M.); eliasdelgado@telefonica.net (E.D.); 10 Laboratorio de Endocrinología, Hospital Universitario San Carlos de Madrid, 28040 Madrid, Spain; elenabordiu@yahoo.es; 11 Diabetes Research Group, Hospital Universitario de Cruces, UPV-EHU, 48903 Baracaldo, Spain; 12 EAP Raval Sud, Institut Català de la Salut, Red GEDAPS, Primary Care, Unitat de Suport a la Recerca (IDIAP e Fundació Jordi Gol), 08001 Barcelona, Spain; 13 Diabetes Unit, Hospital Arnau de Vilanova, 46015 Valencia, Spain; jgirbesb@comv.es; 14 Genotyping and Genetic Diagnosis Unit, Fundación de Investigación del Hospital Clínico de Valencia-INCLIVA, 46010 Valencia, Spain

**Keywords:** dairy, yogurt, milk, diabetes, obesity, hypertension

## Abstract

To date it is not clear what the role of dairy products is in metabolic diseases like diabetes, obesity, and hypertension. Therefore, the aim of this study is to test the association between dairy product consumption and those pathologies. A cross-sectional study was conducted with 5081 adults included in the di@bet.es study, from 100 health centers around Spain. Food frequency questionnaires were carried out concerning consumption habits, which included dairy product consumption. Logistic regression models were used for the association analyses between the variables controlling confounding variables. Women had a higher consumption of milk, cheese, or yogurt than men (*p* < 0.0001), but men consumed more sugar dairy products (*p* < 0.001). People who live in the North of Spain consume more dairy products than those who live in the East. Dairy product consumption was inversely associated with the presence of hypertension regardless of age, sex, geographical region, and body mass index (BMI) (Odds Ratio (OR) 0.743; *p* = 0.022). The presence of obesity was inversely associated with dairy consumption regardless of age, sex, and geographical region (OR 0.61; *p* < 0.001). Milk consumption was not associated with diabetes. Our results show that consuming dairy products is associated with a better metabolic profile in the Spanish population.

## 1. Introduction

Milk products contain proteins that favor the digestion in addition to being proteins of high biological value. They also contain elements which might make their consumption have some favorable effect on certain chronic diseases (fat carbohydrates, vitamins and minerals, calcium, and phosphorus) [1]. However, studies concerning dairy product intake and the risk of type 2 diabetes (T2DM) and hypertension, among other diseases with high cardiovascular risk, have yielded inconsistent results [2].

In Spain, according to current studies, the prevalence in adults of type 2 diabetes is 12.5%, hypertension 39.9%, and obesity 26.6% [3]. Although the majority of the scientific evidence supports the fact that the intake of milk and dairy products may protect against the most prevalent chronic diseases [4,5], there are few nationwide studies that have investigated the relationship between dairy product intake and the prevalence of diabetes, although in general, it seems there is an inverse relationship between both [6].

Nestel et al. [7] demonstrated, in an interesting experiment, that fermented milk, but not skimmed milk, had a clear beneficial effect on a profile of inflammation biomarkers when compared with other types of milk. In addition, the consumption of yogurt and other dairy products was associated with a lower risk of obesity in observational studies; an aspect which has been partially confirmed by clinical trials [8].

Dairy products containing milk fat are a major food source of saturated fat. A high intake of some kinds of saturated fat has been linked to an increased risk of cardiovascular disease [9]. A recent prospective cohort study has concluded that dairy consumption is associated with lower risk of mortality and major cardiovascular disease events in a diverse multinational cohort [5]. Most of the prospective studies show that the replacement of dairy saturated fat with unsaturated fat or whole grains lowers cholesterol and LDL cholesterol [10]; while the majority of clinical trials showed no effects on the plasma lipid profile after low fat dairy product consumption [11,12]. However, several clinical studies have suggested a beneficial effect of dairy product consumption on plasma lipid levels, especially with full-fat natural cheese and fermented strain-specific yogurt products [1].

A meta-analysis of prospective cohort studies suggests that the consumption of dairy products was associated with a reduction in the risk of high blood pressure [13,14,15]; however, other studies reported no differences between blood pressure and the consumption of dairy products [16,17].

A recent meta-analysis [18], which included ten cross-sectional studies, two nested case-control studies, and twenty nine cohort studies, concluded that specific types of dairy food consumption such as milk and yogurt as well as total dairy food consumption were inversely related to the risk of the metabolic syndrome and its components.

To date, it is not clear what the role of dairy products in these pathologies is in the Spanish population as a whole and whether there are adequate studies regarding this issue in this population. There are very few studies in Spain to assess the relationship between dairy products and metabolic diseases. Most of them are in children, adolescents [19] (511 individuals), or elderly people [20] (3454 individuals analyzed) and focus only on cardiovascular disease or refer to specific towns [21] (790 individuals). All these previous studies have suggested that there might be an effect of dairy consumption on metabolic diseases; therefore, we performed this study in a Spanish nationwide sample with the objective of testing the association between dairy product consumption and diabetes, obesity and hypertension in the Spanish adult population.

## 2. Materials and Methods

### 2.1. Population

The di@bet.es study is a national, cross-sectional, population-based survey undertaken between 2009 and 2010 (41.6% men and 58.4% women) with subjects from the entire Spanish population. Briefly, about 100 health centers or their equivalent were selected from all around the country, after which 100 individuals aged ≥18 years were randomly selected from each health center to obtain a random representative sample of the civilian noninstitutionalized Spanish population. A total of 10,227 adults were invited to participate via mail or telephone, of whom 56% (*n* = 5728) attended for examination. Of these, 9.9% were excluded by protocol (institutionalized, severe disease defined as any disorder that prevented the subject from going to the health center and giving informed consent, pregnant or recent delivery) and additionally 1.53% was excluded because of missing data. The final number of subjects studied was 5081. 

The study variables were a clinical and demographic structured survey, physical examination (height, weight, blood pressure, body mass index (BMI), and waist and hip circumference), lifestyle survey, and oral glucose tolerance test (OGTT) (75 g). The study was conducted in accordance with the Declaration of Helsinki, and was approved by the Ethics and Clinical Investigation Committee of Hospital Regional Universitario de Malaga (Ref. SE-037) in addition to other regional ethics and clinical investigation committees all over Spain. Written informed consent was obtained from all the participants.

### 2.2. Variables and Procedures

Participants were invited by mail and/or telephone to attend an examination visit at their health center. Information was collected using an interviewer administered structured questionnaire, followed by a physical examination by a nurse, who prior to the study had undergone a specific training course in order to standardize procedures. The field work was performed by 7 teams each composed of a nurse and a dietician.

The data included are age, sex, and personal history of diabetes and hypertension. 

Subjects with baseline capillary blood glucose levels lower than 7.8 mmol L^−1^ (140 mg dL^−1^) and those not currently receiving treatment for diabetes underwent a standard OGTT. Capillary blood glucose was measured at baseline and 2 h after the OGTT with a Glucometer OneTouch^®^ Ultra^®^ (Lifescan, Madrid, Spain), and a venous blood sample was taken from each subject (overnight fast and post load). Samples were immediately centrifuged and stored at −18 °C (15 days maximum) until transport to the centralized biobank (Ciberdem and Málaga Regional Hospital Biobank, Andalusian Public Health System Biobank) where they were stored at −80 °C for later analysis. 

Serum glucose was measured enzymatically in an Architect C8000 Analyzer (Abbott Laboratories SA, Madrid, Spain). The diagnosis of diabetes was based on the serum glucose levels according to the 1999 World Health Organization (WHO) criteria [22].

Blood pressure was measured using a blood pressure monitor (Hem-703C, Omron, Barcelona, Spain) following the instructions of the manufacturer. Blood pressure was measured with the subject seated and after 5 min of rest. Two readings were obtained and their mean was used in the analyses. Hypertension was defined as ongoing antihypertensive treatment or systolic blood pressure ≥140 and/or diastolic blood pressure ≥90 mmHg [23]. 

Subjects were asked about the usual frequency of consumption of (1) milk, yogurt, or cheese; (2) sugary dairy products (packaged milkshakes, pudding, custard, and ice cream); and (3) butter or cream. The data for the previous 6 months of these three groups of dairy were recorded. The frequencies of consumption of the three groups of dairy were reported on an incremental scale with eleven levels (never or almost never, once per month, 2–3 times per month, once per week, 2–3 times per week, 4–6 times per week, once per day, twice per day, 3 times per day, 4 times per day, and 5 or more times per day). The results were finally recoded in four categories: less than once per day; once per day; twice per day; and 3 or more times per day.

The educational level of all participants was also asked and the information was recorded as a 2 category variable: Mandatory school attendance (younger than 16 years old) or higher education. Weight, height, waist, and hip circumferences were measured in all the participants by standardized methods, and the BMI was calculated as weight/height^2^. A BMI equal to or higher than 30 kg/m^2^ was considered obese [24].

### 2.3. Statistical Study

The hypothesis contrast between proportions was performed using the chi-square test. The strength of associations between one variable (dependent: presence or absence of hypertension, Type 2 Diabetes Mellitus (T2DM), obesity) and other potentially explanatory variables (age, sex, area, dairy product intake, and BMI) was measured by calculating the Odds Ratio OR (95% confidence interval CI), obtained from the coefficients of different logistic regression models. In all cases the level of rejection of a null hypothesis was α < 0.05. Analyses were performed using R statistical software (Department of Statistics, University of Auckland, Auckland, New Zealand).

## 3. Results

Women had a significantly higher consumption of milk, cheese, or yogurt than men; however, men consumed more sugar dairy products (shakes, puddings, and ice cream). Women consumed between two or three dairy products per day (35.9 and 36.5%, respectively), while men often consumed two dairy products per day (34.8%). Butter or cream consumption was similar in both (Table 1).

Most of the participants consumed mainly milk, cheese, or yogurt at least twice a day or more times a day (28–36.4%). Of all of them, people aged between 18 and 30 years consumed sugary dairy products most often, while those over 75 years consumed the least (Table 1).

Butter or cream consumption decreased with age, establishing higher consumption among people aged 18–30 (by 7.6%), whose butter consumption was three or more times a day. This consumption decreased progressively with age, up to 2% in people over 75 years (Table 1).

In the dairy product consumption by geographical area (Table 1), we found that in the east region of Spain only 19.4% of the participants consumed dairy products less than once a day. The consumption of milk, cheese, or yogurt in this region was the lowest compared to their consumption in the rest of the studied areas; however, the consumption of sugary dairy products was higher than in the others (11.8%). The north area was where dairy products were most frequently consumed—46.5% of the participants consumed dairy products three or more times a day—followed by the south region (37.2%). Also, 51.1% of the subjects from the north area never consumed sugary milk products; this was the region where these products were less frequently consumed.

Intake of three or more dairy products (milk, cheese, or yogurt) was inversely associated with the presence of hypertension regardless of age, sex, geographical area, and BMI when all subjects were considered (Table 2), with a significant *p* for trend for the dairy consumption (*p* = 0.02). The association between high dairy intake and the presence of hypertension was close to be statistically significant (*p* = 0.068) after removing those subjects with known diabetes from the model (Table 2); nevertheless, the p for trend for the dairy intake remained significant (*p* = 0.03).

In our study, the consumption of dairy products was also negatively associated with obesity in a logistic regression model adjusted by age, sex, geographical area hypertension, and educational level, even when subjects with known diabetes were removed from the analysis (Table 3). The *p* trend for dairy consumption was statistically significant in both models (*p* < 0.001).

We did not find any association between dairy product intake and the presence of diabetes adjusting by age, sex, geographic area obesity, hypertension, and educational level as confounding factors (Table 4). The inclusion or not in the analysis of those subjects with known diabetes did not modify the lack of association (data not shown) and the *p* trend for dairy products consumption was also not significant in both cases.

Regarding the frequency of dairy product consumption by community, we found a consumption gradation from East to West, being the east area where less dairy products were consumed compared to the northwest area.

## 4. Discussion

According to our results, in Spain, women have a higher consumption of milk, cheese, or yogurt than men, but men consume more sweetened dairy products. The majority of the Spanish population follows the latest consensus published by the Spanish Society of Community Nutrition SSCN [25] regarding recommendations of daily consumption of milk and dairy products, consuming dairy products twice or more times a day: people from north and east Spain present the highest and lowest dairy product consumption, respectively.

In this study we investigated the suggested association between dairy product consumption and metabolic diseases such as diabetes, obesity, and hypertension in the Spanish adult population. Most of the studies that focus on the relationship of dairy intake and diabetes in Spain [19,20,21] suggest an association between them; however, these studies were performed in selected groups of people and they are not nationally representative. Among the studies that analyze the relationship between dairy product intake and glucose metabolism in other populations [26,27,28,29], a recent meta-analysis of prospective cohort studies has concluded that overall, there is scientific evidence of neutral or beneficial associations with T2DM [2]. However, the subgroup analysis in the Soedamh-Muthu et al. meta-analysis also returned the stronger inverse association for dairy and milk consumption in the Asian population, while European populations show null association [2]. Consistent with the results from the European population of the Soedamh-Muthu meta-analysis, in our study, dairy product consumption was not associated with the presence of T2DM. 

Regarding the relationship between dairy intake and obesity, several mechanisms have been proposed by which the consumption of dairy products may influence weight control. Firstly, dairy products reduce lipogenesis and increase lipolysis in adipose tissue. Secondly, high calcium intake can lead to increased fecal fat excretion [30] and has a role in fat oxidation [31]. Fat absorption in the intestine may be inhibited by calcium via binding to saturated fatty acids (SFA) or bile acids. Additionally, the regulation of intracellular calcium levels are also involved in lipogenesis and lipolysis so they can affect fat mass [32,33]. A recent updated meta-analysis including 37 randomized control trials has suggested a beneficial effect of energy-restricted dairy consumption on body weight and body composition; however, in the absence of calorie restriction, a high dairy consumption might lead to an increase in body weight [34]. Alternatively, results from a systematic review and meta-analysis including cohort studies have also suggested the beneficial effects of some dairy products but not total dairy consumption on body weight; however, these results should be cautiously interpreted due to the high heterogeneous risk estimates [35]. Our results are in line with these, suggesting a possible beneficial effect of dairy consumption on body weight, having found an inverse association between dairy product intake and the presence of obesity.

Numerous studies have reported rather mixed effects of dairy consumption on blood pressure. Results from a comprehensive review based primarily on data from meta-analyses of randomized control trials and from individual randomized control trials conducted in disease-free individuals have suggested that there is no apparent risk of potential harmful effects of dairy consumption, irrespective of the content of dairy fat, on blood pressure among other cardiometabolic risk factors [17]. However, systematic reviews and meta-analyses from prospective observational studies report an inverse association between the consumption of dairy products, particularly low-fat, and the risk of hypertension [13,14,15]. In our study, the intake of three or more dairy products per day was inversely associated with hypertension with a statistically significant trend for the dairy serving consumption, and although this association was only close to being significant after removing those subjects with known diabetes from the analysis, the observed trend remained significant. All these results would support the previous cohort studies suggesting a beneficial effect of dairy product consumption on hypertension. These suggested positive effects might be explained by the fact that dairy products, despite their saturated fat content, have other components such as vitamin D, calcium, potassium, and phosphorus, in addition to some bioactive peptides, which might provide some antihypertensive properties such as triggering the synthesis of 1,25-hydroxyvitamin D [33], stimulating lipolysis, and inhibiting lipogenesis [18,32] or inhibiting the function of the angiotensin converting enzyme [32,33]. 

The present study includes those limitations inherent to a cross-sectional study, preventing observations regarding the directions of the associations found. The lack of quantitative nutritional data, or the types of dairy products (skimmed/whole), is an important limitation of this study that should be solved in future investigations performing quantitative or semiquantitative nutritional surveys. Also, the definition of dairy product has been constructed as a combination of the frequency of intake of dairy (milk, cheese, or yogurt) and each of these foods could have different health effects. Nevertheless, the strength of our work is that the study included a large representative sample of the Spanish population which allows us to have global vision of the consumption of dairy products in this population and to determine the effect of these products on metabolic diseases.

## 5. Conclusions

In our study, a higher consumption of dairy products (≥3 servings per day compared with less than once per day) was inversely associated with the presence of hypertension and obesity but no association was observed with the presence of diabetes. There might be an association among dairy product consumption and other metabolic diseases; however, more studies are needed to ascertain its possible effects.

## Figures and Tables

**Table 1:** Consumption of dairy products based on sex, age, and geographical area.

	SEX				AGE						GEOGRAPHICAL AREA					
	All	M	W	*p*	18–30	31–45	46–60	61–75	>75	*p*	S	N	C	N–E	E	*p*
	*n* = 5081	*n* = 2180	*n* = 2901		*n* = 681	*n* = 1453	*n* = 1397	*n* = 1105	*n* = 445		*n* = 1622	*n* = 626	*n* = 1260	*n* = 922	*n* = 651	
**Consumption of dairy (milk, cheese, or yogurt) (%)**																
<once/day	10.3	12.4	8.7	<0.001	14.2	9.9	10.9	8.9	6.7	<0.001	10.8	5.1	7.1	10.6	19.4	<0.001
once/day	21.8	25.6	19.0		23.1	20.2	20.8	24.2	22.7		18.4	17.3	21.9	21.7	34.9	
twice/day	35.4	34.8	35.9		34.7	35.4	34.8	36.2	36.4		33.6	31.2	38.4	39.6	32.3	
≥3 times/day	32.5	27.2	36.5		28	34.4	33.6	30.8	34.2		37.2	46.5	32.5	28.1	13.5	
**Consumption of shakes, puddings, ice cream, etc. (%)**																
never	45.5	40.1	49.5	<0.001	32	40.1	48.7	52	57.5	<0.001	49.1	51.1	42.7	39.4	45	<0.001
<once/day	48.4	52.7	45.3		58	52.8	46.4	43.6	38		42.7	45.2	55.3	55.1	43.2	
≥ once/day	6.1	7.2	5.2		10	7.2	4.9	4.3	4.5		8.2	3.7	2	5.5	11.8	
**Butter consumption (%)**																
<once/day	72.5	73.2	72.0	0.8	63	65	72.7	82.5	86.1	<0.001	74.7	83.7	75.6	61.9	65.1	<0.001
once/day	9.7	9.3	9.9		10.6	12	9.1	8.6	5.2		5.4	5.4	11.5	16.5	11.1	
twice/day	13.3	13.0	13.5		18.8	17.4	14	6.2	6.7		14	7.3	10.5	17.5	16.7	
≥3 times/day	4.6	4.5	4.6		7.6	5.6	4.3	2.6	2		5.9	3.5	2.5	4.1	7.1	

Data represent proportions (%). *p* value for X^2^ test to contrast proportions. Sex (Man = M, Women = W), geographical area (North = N, South = S, East = E, West = W, Center = C).

**Table 2:** Hypertension and dairy product consumption.

	All Subjects (*n* = 5081)			Subjects with Known Diabetes Excluded (*n* = 4538)		
Variables	*p*	OR ^a^	95% CI ^b^	*p*	OR ^a^	95% CI ^b^
Age	<0.001	1.083	(1.07–1.08)	<0.001	1.080	(1.07–1.08)
Sex	<0.001	0.507	(0.44–0.58)	<0.001	0.488	(0.42–0.56)
South area	RC ^c^	1		RC ^c^	1	
North area	<0.001	1.58	(1.24–2.00)	<0.001	1.600	(1.25–2.03)
Center area	<0.001	0.669	(0.55–0.81)	<0.001	0.661	(0.53–0.81)
Northeast area	0.011	0.766	(0.62–0.94)	0.028	0.783	(0.63–0.97)
East area	0.012	0.740	(0.58–0.93)	0.042	0.777	(0.61–0.99)
Dairy < once/day	RC ^c^	1		RC ^c^	1	
Dairy once/day	0.470	0.907	(0.69–1.18)	0.530	0.916	(0.69–1.20)
Dairy twice/day	0.107	0.815	(0.63–1.04)	0.111	0.810	(0.62–1.04)
Dairy 3 times or more	0.022	0.743	(0.57–0.95)	0.068	0.782	(0.60–1.01)
Body mass index (kg m^2^)	<0.001	1.138	(1.12–1.15)	<0.001	2.698	(2.30–3.16)
Mandatory school attendance	RC ^c^	1		RC ^c^	1	
School attendance over 16 years old	0.007	0.81	(0.69–0.94)	0.012	0.816	(0.69-0.95)

Logistic regression model (Dependent variable: Hypertension encoded as 0: No hypertension and 1: hypertension) Adjusted for age, sex, body mass index, geographical area, and educational level. ^a^ OR: odds ratio; ^b^ 95%CI: 95% confidence interval; ^c^ RC: Reference category.

**Table 3:** Obesity and dairy product consumption.

	All Subjects (*n*:5081)			Subjects with Known Diabetes Excluded (*n* = 4538)		
Variables	*p*	OR ^a^	95% CI ^b^	*p*	OR ^a^	95% CI ^b^
Age	0.005	1.007	(1.00–1.01)	0.003	1.008	(1.03–1.03)
Sex	0.92	1.006	(0.88–1.14)	0.814	0.983	(0.85–1.13)
South area	RC ^c^	1		RC ^c^	1	
North area	<0.001	0.535	(0.43–0.66)	<0.001	0.567	(0.44–0.71)
Center area	0.001	0.745	(0.62–0.88)	0.005	0.767	(0.63–0.92)
Northeast area	<0.001	0.708	(0.58–0.85)	0.003	0.736	(0.60–0.90)
East area	0.001	0.685	(0.53–0.85)	0.002	0.695	(0.55–0.87)
Dairy < once/day	RC ^c^	1		RC ^c^		
Dairy once/day	0.02	0.758	(0.60–0.95)	0.049	0.748	(0.60–0.99)
Dairy twice/day	0.006	0.737	(0.59–0.91)	0.04	0.710	(0.56–0.89)
Dairy 3 times or more	<0.001	0.641	(0.51–0.80)	<0.001	0.624	(0.49–0.79)
Hypertension	<0.001	2.9	(2.49–3.37)	<0.001	2.768	(2.36–3.24)
Mandatory school attendance	RC ^c^	1		RC ^c^	1	
School attendance over 16 years old	<0.001	0.61	(0.52–0.71)	<0.001	0.607	(0.52–0.71)

Logistic regression model (Dependent variable: Obesity encoded as 0: No obesity (BMI < 30 kg/m^2^) and 1: obesity (BMI > 30 kg/m^2^)) Adjusted for age, sex, hypertension, geographical area and educational level. ^a^ OR: odds ratio; ^b^ 95%CI: 95% confidence interval; ^c^ RC: Reference category.

**Table 4:** Diabetes and frequency of dairy product consumption.

Variables	*p*	OR ^a^	95% CI ^b^
Age	<0.001	1.053	(1.04–1.06)
Sex	<0.001	0.559	(0.46–0.67)
South area	RC ^c^	1	
North area	0.002	0.616	(0.45–0.83)
Center area	<0.001	0.588	(0.45–0.75)
Northeast area	0.012	0.715	(0.55–0.92)
East area	0.646	0.935	(0.70–1.24)
Dairy < once/day	RC ^c^	1	
Dairy once/day	0.675	1.073	(0.77–1.49)
Dairy twice/day	0.712	1.062	(0.77–1.45)
Dairy 3 times or more	0.801	0.959	(0.69–1.33)
Hypertension	<0.001	2.523	(2.00–3.18)
Body mass index (kg m^-2^)	<0.001	2.064	(1.72–2.47)
Mandatory school attendance	RC ^c^	1	
School attendance over 16 years old	0.196	0.859	(0.46–0.67)

Logistic regression model (Dependent variable: Diabetes encoded as 0: No diabetes and 1: diabetes). Adjusted for age, sex, obesity, and geographical area. ^a^ OR: odds ratio; ^b^ 95%CI: 95% confidence interval; ^c^ RC: Reference category.

## Appendix A. Di@bet.es Study Group

Sergio Valdés (Centro de Investigación Biomédica en Red de Diabetes y Enfermedades Metabólicas Asociadas [CIBERDEM]; Servicio de Endocrinología y Nutrición, Hospital Universitario Carlos Haya, Instituto de Investigación Biomédica de Málaga [IBIMA], Malaga, Spain), Federico Soriguer (CIBERDEM; Servicio de Endocrinología y Nutrición, Hospital Universitario Carlos Haya, Instituto de Investigación Biomédica de Málaga [IBIMA], Malaga, Spain), Albert Goday (Servicio de Endocrinología y Nutrición, Hospital del Mar, Barcelona, Spain), Anna Bosch-Comas (CIBERDEM; Institut d’Investigacions Biomèdiques August Pi i Sunyer [IDIBAPS], Hospital Clínic de Barcelona, Barcelona, Spain), Elena Bordiú (Laboratorio de Bioquímica, Hospital Universitario San Carlos, Madrid, Spain), Alfonso Calle-Pascual (Servicio de Endocrinología y Nutrición, Hospital Universitario San Carlos, Madrid, Spain), Rafael Carmena (CIBERDEM; Departamento de Medicina y Endocrinología, Hospital Universitario de Valencia, Valencia, Spain), Roser Casamitjana (CIBERDEM; Centro de Diagnóstico Biomédico, Hospital Clínic de Barcelona, Barcelona, Spain), Luis Castaño (CIBERDEM; Unidad de Investigación, Hospital Universitario Cruces, Universidad del País Vasco/Euskal Herriko Unibertsitatea [UPV/EHU], Baracaldo, Vizcaya, Spain), Conxa Castell (Servei d’Educació Sanitària, Generalitat de Catalunya, Barcelona, Spain), Miguel Catalá (CIBERDEM; Departamento de Medicina y Endocrinología, Hospital Universitario de Valencia, Valencia, Spain), Elias Delgado (Servicio de Endocrinología y Nutrición, Hospital Central de Asturias, Oviedo, Asturias, Spain), Joseph Franch (Equipo de Atención Primaria Raval Sud, Institut Català de la Salut, Red GEDAPS [Grupo de Estudio de la Diabetes en Atención Primaria de la Salud], Unitat de Suport a la Recerca, Institut d’Investigació en Atenció Primària Jordi Gol, Barcelona, Spain), Sonia Gaztambide (CIBERDEM; Servicio de Endocrinología y Nutrición, Hospital Universitario de Cruces, UPV/EHU, Baracaldo Vizcaya, Spain), Joan Girbés (Unidad de Diabetes, Hospital Arnau de Vilanova, Valencia, Spain), Ramón Gomis (CIBERDEM; IDIBAPS, Hospital Clínic de Barcelona, Barcelona, Spain), Galde Gutiérrez (CIBERDEM; Unidad de Investigación, Hospital Universitario de Cruces, UPV/EHU, Baracaldo, Vizcaya, Spain), Alfonso López-Alba (Sociedad Española de Diabetes, Madrid, Spain), María Teresa Martínez-Larrad (CIBERDEM; Instituto de Investigación Sanitaria del Hospital Clínico San Carlos [IdISSC], Madrid, Spain), Edelmiro Menéndez (Servicio de Endocrinología y Nutrición, Hospital Central de Asturias, Oviedo, Asturias, Spain), Inmaculada Mora-Peces (Servicio Canario de Salud, Tenerife, Spain), Emilio Ortega (CIBERDEM; IDIBAPS, Hospital Clínic de Barcelona, Barcelona, Spain), Gemma Pascual-Manich (CIBERDEM, Spain), Manuel Serrano-Rios (CIBERDEM; IdISSC, Madrid, Spain), Inés Urrutia (CIBERDEM; Unidad de Investigación, Hospital Universitario de Cruces, UPV/EHU, Baracaldo, Vizcaya, Spain), Jose Antonio Vázquez (CIBERDEM; Servicio de Endocrinología y Nutrición, Hospital Universitario de Cruces, UPV/EHU, Baracaldo, Vizcaya, Spain), Joan Vendrell (CIBERDEM; Servicio de Endocrinología y Nutrición, Hospital Universitario Joan XXIII, Institut d’Investigacions Sanitàries Pere Virgili, Tarragona, Spain) y Gemma Rojo-Martínez (CIBERDEM; Servicio de Endocrinología y Nutrición, Hospital Universitario Carlos Haya, IBIMA, Malaga, Spain).
